# Determining Pro-Oxidant Antioxidant Balance (PAB) and Total Antioxidant Capacity (TAC) in Patients with Schizophrenia 

**Published:** 2018-07

**Authors:** Farnaz Zahedi Avval, Niloufar Mahmoudi, Abolfazl Nosrati Tirkani, Lida Jarahi, Daryoush Hamidi Alamdari, Seyed Alireza Sadjadi

**Affiliations:** 1 Department of Clinical Biochemistry, School of Medicine, Mashhad University of Medicine Sciences, Mashhad, Iran.; 2 Metabolic Syndrome Research Center, Mashhad University of Medicine Sciences, Mashhad, Iran.; 3 Department of Psychiatry, School of Medicine, Mashhad University of Medicine Sciences, Mashhad, Iran.; 4 Department of Community Medicine, Mashhad University of Medicine Sciences, Mashhad, Iran.; 5 Psychiatry and Behavioral Sciences Research Center, Ibn-e-Sina Hospital, School of Medicine, Mashhad University of Medicine Sciences, Mashhad, Iran.

**Keywords:** *Pro-Oxidant Antioxidant Balance*, *Reactive Oxygen Species*, *Schizophrenia*, *Total Antioxidant Capacity*

## Abstract

**Objective:** Schizophrenia is a disease with unknown etiology. There is evidence suggesting that oxidative damage occurs in schizophrenia. Oxidative damage may arouse from imbalance between oxidant and anti-oxidant factors in cellular and tissue environment. Although it may not be the primary cause, it can worsen the disease and may be a reason of poor response to therapy in these patients. The present study aimed at evaluating the pro-oxidant antioxidant balance (PAB) and total antioxidant capacity (TAC) in serum of schizophrenia patients. PAB is an assay to determine the pro-oxidant load and antioxidant capacity in a single measurement.

**Method** **:** In this cross- sectional study, patients with diagnosis of schizophrenia, who referred to a psychiatry university hospital (Ibn-e-Sina Hospital) affiliated to Mashhad University of Medical Sciences, were enrolled. Patients' demographic characteristics and laboratory data were recorded from patients’ files. Serum PAB and TAC were measured using a special PAB assay and commercial kit, respectively. Data were analyzed using SPSS 16.

**Results:** A total of 84 individuals (42 schizophrenia cases and 42 healthy controls) participated in this study. Controls were age and sex-matched with the patients’ group. The mean TAC in the patient and control groups was 0.49±0.04 and 0.51±0.04 nmol/L, respectively (p = 0.16). PAB was higher in patients’ group than in controls (127.36±36.44 vs. 118.93±52.34 HK), however, this difference was not statistically significant (p = 0.09). The change was correlated with the chronicity of the disease.

**Conclusion: **Pro-oxidant antioxidant balance was elevated in serum of patients with schizophrenia. These data suggested the occurrence of oxidative stress during the progression of the disease. Lower antioxidant capacity might suggest that patients with schizophrenia could be more susceptible to oxidative stress damage.

Schizophrenia (SZ) is a devastating mental disorder characterized by abnormal behavior and deterioration in social functions and attitudes (1). SZ is a chronic and severe condition which affects probably ~1% of the global population (2). Consumption of alcohol, drug abuse, and malnutrition are classified as environmental risk factors (3). 

Prenatal and neonatal infections, interactions during birth, and maternal malnutrition are classified as genetic risk factors (4). Although SZ is described as one single disease, the involved patients exhibit different response to the same therapy. 

Although the exact etiology of SZ has not been fully understood, it is thought that SZ is a complicated neurodevelopmental disorder that might be affected by many underlying factors. Among the under investigation factors, oxidative stress might be an underlying mechanism associated with disease procession (5, 6). 

Disturbance of pro-oxidant antioxidant balance is usually described as the oxidative stress which takes part in the pathogenesis of various neurocognitive diseases, autoimmune disorders, and cancer (7, 8). Oxidants are produced during either normal metabolism or originate from environmental factors. Reactive oxygen species (ROS) and reactive nitrogen species (RNS), along with ultra-violet radiation, air pollution, alcohol, and smoking are the most prevalent causes of oxidative damages in patients suffering from SZ. The enzymatic antioxidant network consists of catalase (CAT), glutathione peroxidase (GPX), superoxide dismutase (SOD); moreover, non-enzymatic antioxidants include vitamin A, C, and E, bilirubin, flavonoid, and ubiquinone, which react as the oxidant detoxifying mechanism (9). 

Clinical investigations have shown the significant alterations in enzymatic and/or non-enzymatic antioxidants in the serum of patients with SZ (10, 11). Several studies revealed the raised level of oxidative damage not only due to reduced antioxidants but also increased pro-oxidants and free radicals (12). ROS changes the permeability and fluidity of phospholipids in plasma membrane and may result in impaired signaling pathways (12). The blockage of N-Methyl-D-aspartate glutamate receptor by phencyclidine is the biochemical basis of glutamate malfunction hypothesis (13). The psychosis-inducing effect of dopaminergic agonists occurs due to dopamine network dysfunction (14, 15). In addition to the elevated level of lipid peroxidation metabolites and nitric oxide (NO) generation, lower activity of superoxide dismutase (SOD), catalase (CAT), and glutathione peroxidase (GPX) have been reported in SZ (16). Moreover, it has been shown that glutathione (GSH), a key factor in the protection of CNS, is significantly decreased in cerebrospinal fluid (CSF) of patients with schizophrenia (17). 

Identifying substances that are involved in the damage of the neurons and relapse of schizophrenia is of high clinical importance. Evaluation of abundant metabolites altered by oxidative stress process is an expensive, time-consuming, and imprecise procedure (18). Despite the existing data, still new approaches are needed, particularly with potential clinical applications in oxidant/antioxidant assays. One such assay is pro-oxidant antioxidant balance (PAB) technique. The assay is a new strategy to determine the pro-oxidant load and antioxidant capacity in a single assay (19). The assay provides general view on the oxidant/antioxidant status of the patients in one single experiment. In the present study, both PAB assay and TAC measurement were performed in SZ and control groups to determine the oxidative stress status to give a general view of oxidative stress.

## Materials and Methods

***Patients and Samples***

The present study was conducted on a group of 42 Patients with SZ selected according to the DSM-IV-TR criteria in the Ibn-e-Sina psychiatry university hospital affiliated to Mashhad University of Medical Sciences, Mashhad, Iran, during October 2014 and October 2016. Healthy controls were 42 age and sex-matched volunteer participants. A questionnaire addressing history of any diagnosed clinical conditions including diabetes mellitus, inflammatory disease, and cardiovascular disease was filled for each participant. 

The exclusion criteria were other psychiatric disorders, pregnancy, electroconvulsive therapy (ECT) during the last year, consumption of vitamin supplements, and age under 18 or over 60 years. Blood samples were collected and sera were kept at -80°C until analysis. 

***Determinatining Total Antioxidant Capacity (TAC)***

TAC was measured by a chemical colorimetric method using a commercial antioxidant assay kit purchased from Cayman Chemical Company, USA (Cat. Number: 709001). This method relies on the ability of antioxidants in the sample to inhibit the oxidation of ABTS® (2, 2’-azino-di- [3-ethylbenzthiazoline sulphonate]) to ABTS® •+ using metmyoglobin (20).

***Pro-oxidant Antioxidant Balance (PAB) Assay***

Determining all oxidants and antioxidants separately is time- consuming and costly. Different methods have been developed to calculate the total oxidants and antioxidants. The PAB assay is a test to determine the oxidants and antioxidants simultaneously in one single test (21). Briefly, the standard solution was prepared by mixing different proportions (0-100%) of 250 µM hydrogen peroxide with 3 mM uric acid in 10 mM sodium hydroxide. Then, in 10 mL of dimethyl sulphoxide (DMSO) and 60 mg of tetramethylbenzidine (TMB) powder was dissolved. To prepare TMB+, 400 µL of TMB dissolved in DMSO was added to 20 mL of 0.05 M acetate buffer with pH = 4.5. In the next step, 70 µL of 100 mM chloramine T fresh solution was added, shacked, and incubated in a dark place for 2 hours at room temperature. Then, 25 U of peroxidase enzyme solution was added to TMB+, aliquoted into 1 mL volume, and kept at -20ºC. The TMB solution was prepared by blending 200 µL TMB dissolved in DMSO in 10 mL of 0.05 M acetic acid (pH = 5.8). To prepare the working solution, 1 mL TMB+ and 10 mL TMB solution was incubated in dark place for 2 minutes at room temperature and used directly.

In each well of ELISA plate, 10 µL of each patient's sera, standard or blank (distilled water was used as blank), was well mixed with 200 µL working solution. Then, the plate was incubated for 12 minutes in a dark place at 37ºC. After the end of the incubation time, 100 µL of 2N HCl was added to each well. The optical density (OD) was evaluated at 450 nm while the reference wavelength was 620 nm or 570 nm. The standard curve was prepared by the values determined in standard samples.

The values of PAB assay were expressed in arbitrary HK (Hamidi-Koliakos) unit based on the percentage of hydrogen peroxide evaluated in standard solution. Finally, the patients PAB values were determined according to the prepared standard curve. 

*Ethical Considerations*

This study was reviewed and approved by the ethical committee of Mashhad University of Medical Sciences and was performed based on the ethical codes of Declaration of Helsinki (Ethical code: IR.MUMS.REC.1394.243). A written informed consent was obtained from each participant.

*Statistical Analysis*

The statistical package for the social sciences (SPSS) 16.0 (SPSS Inc., Chicago, IL, USA) was used for statistical analysis, and statistical significance was defined as p<0.05. Comparisons of nominal variables were performed by chi-square and fisher's exact test.

## Results

The patients’ group consisted of 25 males (60%) and 17 females (40%). Among them, 19 individuals (45.2%) had their first episode, while 23 (54.8%) had a history of previous admissions due to multiple episodes of the disorder. Healthy controls with the same number of males and females were matched to the patients group by their age (33.9±6.5 and 33.2±6.9; respectively (p = 0.65)). Total antioxidant capacity in the patients and the control groups was 0.49±0.04 and 0.51±0.04nmol/L, respectively (p = 0.16). The mean of pro- oxidant antioxidant balance (PAB) was higher in patients than controls (127.66 versus 118.93 HK) although the difference was not statistically significant (p = 0.09) (Table 1).

As demonstrated in Table 2, the mean TAC in the first episode patients was higher than in patients with multiple episodes (chronic) although such difference was not statistically significant. The mean value of PAB was also a bit higher in multiply admitted patients although the difference was not statistically significant (Table 2).

**Table 1:** The Measured TAC (Total Antioxidant Capacity) and PAB (Pro-Oxidant Antioxidant Balance) in the Serum of Patients with Schizophrenia Compared to the Healthy Controls

	**Patients**	**Healthy Control**	**P- value**
TAC (nmol/L) (Mean ± SD)	0.49±0.04	0.51±0.04	0.16
PAB (HK†) (Mean ± SD)	127.66±36.44	118.93±25.34	0.09

† Hamidi-Koliakos Arbitrary Unit Based on the Percentage of Hydrogen Peroxide Evaluated in Standard Solution

**Table 2:** The Mean and Standard Deviation of Measured TAC (Total Antioxidant Capacity) and PAB (Pro-Oxidant Antioxidant Balance) in the Serum of Patients Based on the Duration of the Disease in the 2 Groups of Schizophrenia Patients with First Episode or Multiple Episodes

	**First Episode**	**Multiple Episodes**	**P- value**
TAC (nmol/L) (mean ± SD)	0.50±0.03	0.48±0.05	0.70
PAB (HK†) (mean ± SD)	125.2±35.00	129.6±38.20	0.37

† Hamidi-Koliakos Arbitrary Unit Based on the Percentage of Hydrogen Peroxide Evaluated in Standard Solution

## Discussion

In this study, we found that the level of PAB in patients with SZ was slightly elevated compared to the healthy controls. Several studies assessed the level of oxidants and pro-oxidants in patients with SZ. Most of them noted that the level of oxidants increased due to a decrease in antioxidants (22, 23). There is a wide range of pro-oxidants and free radicals in the brain such as nitric oxide synthase, xanthine oxidase, and monoamine oxidase. High levels of enzymatic and non-enzymatic antioxidants protect the neural cells against oxidative damage (24). Despite controversial results in the level of serum antioxidants and activity of catalase, superoxide dismutase and glutathione peroxidase elevated malondialdehyde (MDA), showing an increase in the lipid peroxidation in patients with SZ. An elevated level of lipid peroxidation confirms the oxidative stress and neural damage in CNS (24). 

We used a powerful technique, PAB assay, which provides data on oxidant/antioxidant imbalance status in patients with SZ. Rather high level of PAB revealed that higher level of oxidants, compared to antioxidants, exists in these patients. Although our results were not statistically significant, higher level of PAB showed that oxidative stress occurred in patients with SZ. The oxidative damage occurred slowly over time, causing more profound destruction in neural cells (25). In this study, PAB assay and TAC were performed for patients with first episode and multiple episodes (chronic) of schizophrenia. The results of PAB assay were rather higher in multiple episodes patients compared to the first episode cases. The TAC also slightly decreased in SZ along with multiple episode exacerbations. 

The observed differences were not statistically significant; however, a larger sample size might reveal a hidden significant difference. Such significant difference has been reported by other investigations. For example, it has been reported that patients with chronic SZ might be more affected by oxidative damage than patients with first episode of the disease (26). Also, Boškovic et al. reported a correlation between oxidative stress and chronic phase of SZ (12). In addition, a study conducted in turkey reported that oxidative stress correlates with severity of SZ (27). Patients with schizophrenia are prone to cigarette smoking, addiction, low hygiene, and poor diet, which all might increase their oxidation stress. A study in the USA in 2016 revealed that drug abuse, particularly alcohol, cocaine, and heroin increases the oxidative damage in SZ (28). 

## Limitation

The main limitation of the present study was that the antioxidants were not measured. The participants were not followed up, so the long-term effects on the severity of the disorder was not assessed. It is likely that the oxidative indicators fluctuated across different states of the disease. Thus, a similar study with a larger sample size is highly recommended, especially with consideration of the benefit of prescribing antioxidant or vitamin supplements to manage SZ. Identifying new strategies could improve antioxidant system, prevent neuronal damage, and could ultimately reduce symptoms or prevent recurrences of schizophrenia.

## Conclusion

Since the antioxidant capacity in different communities is different due to diet and culture, in the present study, we aimed at applying a new technique on our patients. Pro-oxidant antioxidant balance (PAB) was rather elevated in serum of patients with schizophrenia. These observations may suggest further clinical investigation to assess the effect of correction of oxidative stress with an adjuvant therapy to manage patients with SZ.
